# Comparative analysis of high-sensitivity cardiac troponin I and T for their association with coronary computed tomography-assessed calcium scoring represented by the Agatston score

**DOI:** 10.1186/s40001-017-0290-9

**Published:** 2017-11-16

**Authors:** Jonas Rusnak, Michael Behnes, Thomas Henzler, Nadine Reckord, Nils Vogler, Mathias Meyer, Ursula Hoffmann, Michele Natale, Julia Hoffmann, Sonja Hamed, Kathrin Weidner, Siegfried Lang, Agnibh Mukherji, Holger Haubenreisser, Stefan O. Schoenberg, Martin Borggrefe, Thomas Bertsch, Ibrahim Akin

**Affiliations:** 1 First Department of Medicine, University Medical Center Mannheim (UMM), Faculty of Medicine Mannheim, University of Heidelberg, Theodor-Kutzer-Ufer 1-3, 68167 Mannheim, Germany; 2 DZHK (German Center for Cardiovascular Research) Partner Site Mannheim, Mannheim, Germany; 3 Institute of Clinical Radiology and Nuclear Medicine, University Medical Center Mannheim (UMM), Faculty of Medicine Mannheim, University of Heidelberg, Mannheim, Germany; 4 Institute of Clinical Chemistry, Laboratory Medicine and Transfusion Medicine, General Hospital Nuremberg, Paracelsus Medical University, Nuremberg, Germany; 5 Department of Internal Medicine III, University Hospital Heidelberg, Faculty of Medicine Heidelberg, University of Heidelberg, Heidelberg, Germany

**Keywords:** Agatston score, Troponin, High-sensitivity troponin, Coronary artery disease, Biomarker

## Abstract

**Background:**

This study evaluates the association between high-sensitivity cardiac troponin I (hs-cTnI) and T (hs-cTnT) and coronary calcium concentration (CAC) detected by coronary computed tomography (CCT) and evaluated with the Agatston score in patients with suspected coronary artery disease (CAD).

**Methods:**

Patients undergoing CCT during routine clinical care were enrolled prospectively. CCT was indicated for patients with a low to intermediate pretest probability for CAD. Within 24 h of CCT examination, peripheral blood samples were taken to measure cardiac biomarkers hs-cTnI and hs-cTnT.

**Results:**

A total of 76 patients were enrolled including 38% without detectable CAC, 36% with an Agatston score from 1 to 100, 17% from 101 to 400, and 9% with values ≥ 400. hs-cTnI was increasing alongside Agatston score and was able to differentiate between different groups of Agatston scores. Both hs-cTn discriminated values greater than 100 (hs-cTnI, AUC = 0.663; *p* = 0.032; hs-cTnT, AUC = 0.650; *p* = 0.048). In univariate and multivariate logistic regression models, hs-cTnT and hs-cTnI were significantly associated with increased Agatston scores. Patients with hs-cTnT ≥ 0.02 µg/l and hs-cTnI ≥ 5.5 ng/l were more likely to reveal values ≥ 400 (hs-cTnT; OR = 13.4; 95% CI 1.545–116.233; *p* = 0.019; hs-cTnI; OR = 8.8; 95% CI 1.183–65.475; *p* = 0.034).

**Conclusion:**

The present study shows that the Agatston score was significantly correlated with hs cardiac troponins, both in univariable and multivariable linear regression models. Hs-cTnI is able to discriminate between different Agatston values. The present results might reveal potential cut-off values for hs cardiac troponins regarding different Agatston values.

*Trial registration* Cardiovascular Imaging and Biomarker Analyses (CIBER), NCT03074253 https://clinicaltrials.gov/ct2/show/record/NCT03074253

## Background

The development of atherosclerosis in coronary arteries requires a well-functioning diagnostic algorithm in clinical practice. With the development of high-sensitivity (hs) cardiac troponin assays, not only the diagnosis of symptomatic coronary artery disease (CAD) but also the detection of subclinical CAD is possible [1–3]. However, European guidelines yet do not recommend a general use of hs cardiac troponins as a risk marker [4].

Nevertheless, there are a number of patients with low to intermediate risk for CAD, who still develop significant lesions in the coronary system. One option in the diagnostic algorithm of patients with low to intermediate risk is the estimation of coronary artery calcium (CAC) by coronary computed tomography (CCT), which is capable of reclassifying patients with an intermediate risk for CAD [5, 6]. Furthermore, with their ability to detect calcium within the investigated tissue [7], CT scanners can quantify the amount of calcium within atherosclerotic lesions [8–10]. Being not present in normal vessel wall, calcifications of the coronary arteries are an indicator for manifest atherosclerosis [11, 12] and the amount of calcium is being known to represent plaque burden and is associated with the extension of atherosclerosis [13–17]. Moreover, CAC has been evaluated as a valuable risk predictor for adverse coronary events such as myocardial infarction and cardiac death in asymptomatic patients [18, 19] and is capable of predicting coronary events beyond other non-invasive tests and risk scores [5].

The Agatston score is the most commonly used and best evaluated score in clinical routine to objectivy CAC. Detrano et al. demonstrated in a multi-ethnic cohort of individuals without known CAD that the Agatston score is a strong predictor of incident coronary heart disease in a follow-up of 3.8 years [20]. Calcium scores under 100 are unlikely to be associated with severe stenosis on coronary angiography and represent a very low risk for obstructive CAD [21, 22]. Furthermore, a large prospective multiethnic study demonstrated that most coronary events such as myocardial infarction or death from CAD occurred in patients with an Agatston score greater than 100 [23].

Only few studies evaluated associations of hs-cTn and CAC screening. In a large Danish study cohort of clinical healthy subjects, an association of hs-cTnI and coronary calcium score was demonstrated [24]. Adding hs-cTnI to the Heart-Score led to a significant increase in discriminative C-statistics for predicting coronary artery calcification [24]. Regarding hs-cTnT, a close correlation of hs-cTnT and the Agatston score in patients without known CAD presenting with chest pain was demonstrated [25].

This study aims to evaluate whether there is an association between hs cardiac troponin I and T concentrations and CAC screening being assessed by CCT using the Agatston score in symptomatic patients with low to intermediate risk for CAD.

## Methods

### Study population and patient selection criteria

The “Cardiovascular Imaging and Biomarker Analyses” (CIBER) study (clinicaltrials.gov identifier: NCT03074253) represents a clinically prospective, controlled, and monocentric study conducted at the University Medical Center Mannheim (UMM), Germany. The research adhered to the principles outlined in the Declaration of Helsinki and was approved by the regional ethics commission II of the Faculty of Medicine Mannheim, University Heidelberg, Germany. Written informed consent was obtained from all patients.

For the present study, consecutive patients undergoing CCT during routine clinical care were included prospectively within the period from January 2015 to August 2015 at the University Medical Centre Mannheim (UMM), Germany. All patients were indicated for CCT due to a low to intermediate PTP of 15–50% presenting with typical or atypical angina pectoris. Exclusion criteria comprised patients with acute myocardial infarction, severe chronic kidney disease being defined as a glomerular filtration rate below 40 ml/min as well as patients with aortic valve stenosis.

### Coronary computed tomography (CCT)

All patients were examined using 2 × 192 slice third-generation dual-source CT system (Force; Siemens Healthineers, Forchheim, Germany). All patients underwent a non-contrast-enhanced cardiac CT for the evaluation of coronary calcifications using the Agatston method. The CCT technique was chosen individually for each patient depending on heart rate and/or rhythm and body mass index, with the goal of minimizing radiation exposure while maximizing diagnostic image quality (mAS range 46–258 mAs). Image acquisition techniques included prospective electrocardiographic (ECG) triggering and prospectively ECG-triggered high-pitch spiral acquisitions. Tube voltage was selected using anatomic-based automated tube voltage selection with a range from 70 to 120 kV (Care kV, Siemens Healthineers, Forchheim, Germany) in combination with automated tube current modulation. In the absence of contraindications, patients received 0.4 mg of sublingual nitroglycerin before image acquisition. B-blockers (5–20-mg intravenous metoprolol tartrate) were used to lower heart rates to less than 65 beats/min in patients undergoing prospectively ECG-triggered high-pitch spiral acquisitions.

Non-contrast-enhanced calcium scoring studies were reconstructed at a section thickness of 3 mm using a dedicated algorithm (Qr36 Siemens Healthineers, Forchheim, Germany). The calcium score was calculated using dedicated software according to the Agatston method [10].

### CCT data analysis

All CT studies were evaluated on a 3D workstation (Multimodality Workplace, Syngo Via Siemens Healthineers Forchheim, Germany) using standard MPR as well as centerline curved MPR. Observers were blinded for biomarker levels. In the first step, the number of plaques that could be identified on CT images in the correct location was recorded to assess the sensitivity of different atherosclerotic plaque types. Those plaques, which were found on CT, were further analyzed.

### Calcium score calculation (Agatston method)

Plaque analysis was performed offline using dedicated software (Syngo VA21, Circulation Plaque Analysis; Siemens Healthineers). Window level and width were determined using a standard window-level setting. The study population was divided into four different groups—patients who did not show any sign coronary calcification (Agatston score = 0), patients with Agatston score from 1 to 100, patients with a score from 101 to 400, and patients with values greater than 400.

### Blood sampling procedures and bio chemical analyses

Within 24 h before or after the CCT, peripheral venous blood samples were taken from each patient, collected in serum monovette^®^ tubes, and centrifuged at 2500×*g* for 10 min at 20 °C. The aliquoted samples were cooled down with liquid nitrogen before being stored at − 80 °C until analysis. The whole processing took part within 2 h after blood extraction. After thawing, the samples were mixed gently by inverting and centrifuged at 2500×*g* for 10 min at 20 °C for cardiac troponin T and NT-proBNP (N-terminal pro-brain natriuretic peptide) analysis. Cardiac troponin T was measured with the cardiac troponin T hs STAT assay on a cobas e602 analyzer (Roche Diagnostics, Mannheim, Germany). The limit of blank (LoB) for this assay was 0.003 µg/l and the limit of detection (LoD) 0.005 µg/l as described in the instructions for use [26]. For TnI measurement, the samples were gently mixed by inverting after thawing and centrifuged for 30 min at 3000×*g* at 4 °C. Cardiac troponin I was measured with the STAT high-sensitivity cardiac troponin I assay on an Architect i1000 analyzer (Abbott, Wiesbaden, Germany) with a LoB of 0.7–1.3 ng/l and a LoD of 1.1–1.9 ng/l [27]. NT-proBNP was measured with the proBNP II STAT assay on a cobas e602 analyzer (Roche Diagnostics, Mannheim, Germany). The LoD for this assay was 5 ng/l [28]. Creatinine, cholesterol, LDLC, HDLC, triglycerides, and uric acid were measured on the cobas c702 analyzer (Roche Diagnostics Mannheim, Germany). All biomarkers were measured in patients’ serum.

### Statistical analysis

Data were analyzed using the software IBM SPSS version 22.0. Categorical variables are expressed as absolute numbers and percentage, whereas continuous variables are shown as mean and range. All biomarkers are presented as the median and interquartile range or mean and standard deviation. For univariate correlations, we used the Spearman Rho test. To analyze the relation between CAC groups and biomarkers, the non-parametric Kruskal–Wallis test was used because the values did not show a normal distribution. To test the presence of a Gaussian distribution, the Kolmogorov–Smirnov test was applied. Receiver operating characteristic (ROC) curves with the area under the curves (AUC) were generated to associate hs-cTn and NT-proBNP with groups of patients with different CAC scores. Multivariate regression models were calculated with backward elimination (Forrest plot). The odds ratios for hs-cTn and cardiovascular risk factors were calculated by binomial logistic regression. All analyses were considered significant when *p* was < 0.05, a statistical trend corresponding to *p* < 0.1.

## Results

### Baseline characteristics

A total of 76 patients were included prospectively with their baseline characteristics being outlined in Table 1. Mean age was 58 years and gender was distributed evenly. About one sixth of patients suffered from diabetes mellitus (16%) and a forth had a known cardiac family history (26%). Nearly a third of study patients (30%) fell into the “adiposity” category, defined as BMI greater than 30 kg/m^2^. Arterial hypertension, hypercholesterolemia, and smoking were the most common risk factors. A minor part of patients suffered from atrial fibrillation.

**Table 1:** Baseline characteristics of patients undergoing CAC screening by coronary CT (*n* = 76)

Characteristics	Value
Age, mean (SD)	58.4 (11.36)
Gender, *n* (%)	
Male	38 (50)
Female	38 (50)
Cardiovascular risk factors, *n* (%)	
Arterial hypertension	39 (51)
Hypercholesterinemia	30 (40)
Cardiac family history	20 (26)
Smoking	24 (32)
Diabetes mellitus	12 (16)
Adiposity	23 (30)
Laboratory parameters, mean (SD)	
Creatinine µmol/l	81.0 (58.9)
Total cholesterol, mmol/l	5.1 (0.95)
LDL cholesterol, mmol/l	3.4 (0.91)
HDL cholesterol, mmol/l	1.4 (0.43)
Triglyceride, mmol/l	1.6 (0.96)
Uric acid, µmol/l	299.9 (78.4)
Medical history, *n* (%)	
Atrial fibrillation	6 (7.9)
Paroxysmal	4 (5.3)
Persistent	1 (1.3)
Permanent	1 (1.3)
COPD	3 (3.9)
Asthma	1 (1.3)
Cancer	15 (19.7)
Radiotherapy	2 (3)
Agatston score	
0	29 (38)
1–100	27 (36)
101–400	13 (17)
> 400	7 (9)

*COPD* chronic obstructive pulmonary disease, *SD* standard deviation

38% revealed an Agatston score of 0, whereas 36% showed a score from 1 to 100, 17% from 101 to 400, and 9% had an Agatston score greater than 400. In the present cohort, measurable concentrations of hs-cTnI were present in all patients (100%) and of hs-cTnT in 72% (55/76) of the study patients.

### Hs cardiac troponins increase alongside increasing Agatston score

**Fig. 1:**
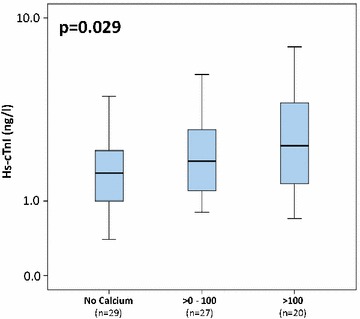
Boxplots illustrating significantly increasing levels of hs-cTnI in the following groups: no CAC, Agatston score from 1 to 100, and Agatston values greater than 100

As presented in Fig. 1, concentrations of hs-cTnI were increasing with increasing Agatston score and were able to differentiate significantly between the different groups of patients without calcification, patients with Agatston score from 0 to 100, and those with Agatston score greater than 100 (*p* = 0.029). The association of hs-cTnI and the groups of patients with Agatston values of 0, values less than 100, values from 100 to 400, and values greater than 400 showed a statistical trend toward significance (*p* = 0.065).

**Fig. 2:**
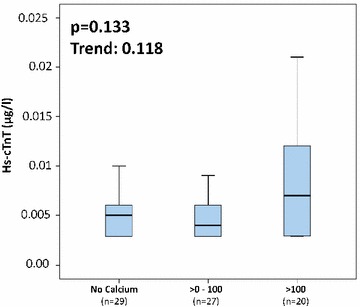
Boxplots illustrating the levels of hs-cTnT in the following groups: no CAC, Agatston score from 1 to 100, and Agatston values greater than 100

Accordingly, hs-cTnT revealed a numerical increase between the different groups of Agatston scoring, as demonstrated in Fig. 2.

### Cardiovascular risk factors and biomarkers according to Agatston categories

As outlined in Table 2, slight increases of hs-cTnI and hs-cTnT were already detectable in patients without measurable calcification (*n* = 29; hs-cTnI, median = 1.6 ng/l; IQR [1.0–2.2 ng/l]; hs-cTnT, median = 0.005 µg/l; IQR [0.0029–0.006 µg/l]). Highest hs-cTnI levels were found in patients with Agatston score greater than 400 (*n* = 7; hs-cTnI, median = 2,5 ng/l; IQR [1.2–7.4 ng/l]), followed by decreasing levels in patients with Agatston score between 101 and 400 (*n* = 13; hs-cTnI, median = 2.3 ng/l; IQR [1.8–3.7 ng/l]) and with Agatston score from 1 to 100 (*n* = 27; hs-cTnI, median = 1.9 ng/l; IQR [1.2–3.1 ng/l]). In contrast to hs-cTnI, hs-cTnT was not increasing according to increasing Agatston values.

**Table 2:** Troponin values according to the degree of Agatston score

	0	≥ 1–100	> 100–400	> 400
*n*	29	27	13	7
hs-cTnT, µg/l, median (IQR)	0.005 (0.0029–0.006)	0.004 (0.0029–0.006)	0.007 (0.003–0.012)	0.007 (0.0029–0.026)
hs-cTnI, ng/l, median (IQR)	1.6 (1.0–2.2)	1.9 (1.2–3.1)	2.3 (1.8–3.7)	2.5 (1.2–7.4)

*IQR* interquartile range, *hs-cTnT* high-sensitivity cardiac troponin T, *hs-cTnI* high-sensitivity cardiac troponin I

Regarding the different Agatston categories, the average age as well as the level of uric acid within the groups is increasing with according to increasing Agatston values. In contrast, LDL decreased according to increasing Agatston values. Patients with evidence of CAC assessed by CCT were more frequently male and smokers. Notably, the group of patients with Agatston values > 400 show suffered from arterial hypertension (86%) and revealed a cardiac family history (57%) compared to patients of the remaining groups. In contrast, none of patients with Agatston values above 400 suffered from diabetes. Accordingly, rate of diabetes was also low in patients without CAC (10%) and in patients with Agatston values from 1 to 100 (11%). However, in the group of Agatston values ranging from 101 to 400 nearly half of patients had diabetes as a preexisting condition.

### Univariable and multivariable linear regression models evaluating the association with the Agatston score

As presented in Table 3, the Agatston score correlated significantly with both hs-cTnI and hs-cTnT as well as with patients’ age and uric acid (*p* < 0.05). No significant correlations were found between Agatston score and NT-proBNP.

**Table 3:** Univariable and multivariable associations on with logarithm of Agatston score

	Univariable		Multivariable hs-cTnT					Multivariable hs-cTnI				
	*r*	*p*	*B*	Cl of *B*	*β*	*t*	*p*	*B*	Cl of *B*	*β*	*t*	*p*
hs-cTnT, μg/l	0.265	0.021	38,451	27,561–4934	1.348	7.046	*0.0001*	–	–	–	–	–
hs-cTnI, ng/l	0.246	*0.032*	–	–	–	–	–	50	24–68	1.643	4.644	*0.0001*
NT-proBNP, ng/l	0.975	*0.004*	0.008	0.195–0.212	0.016	0.083	0.934	0.127	0.074–0.328	0.244	1.259	0.213

*hs-cTnT* high-sensitivity cardiac troponin T, *hs-cTnI* high-sensitivity cardiac troponin I, *NT-proBNP* N-terminal pro-brain natriuretic peptide, *CI* confidence interval

*p* < 0.05 indicates statistical significance

Beyond these univariable associations, both hs-cTns were still significantly associated with the Agatston score after adjusting for all of these risk factors in multivariable linear regression models (Table 3). These models comprised thirteen clinical known risk factors for CAD, such as age, gender, creatinine, uric acid, cholesterol, LDLC, HDLC, BMI, triglycerides, arterial hypertension, cardiac family history, smoking, diabetes and NT-proBNP [hs-cTnT (*β* = 1.348; *T* = 7.046; *p* = 0.0001), and hs-cTnI (*β* = 1.643; *T* = 4.644; *p* = 0.0001)].

### Both hs-cTn discriminate between Agatston score values greater than 100

**Fig. 3:**
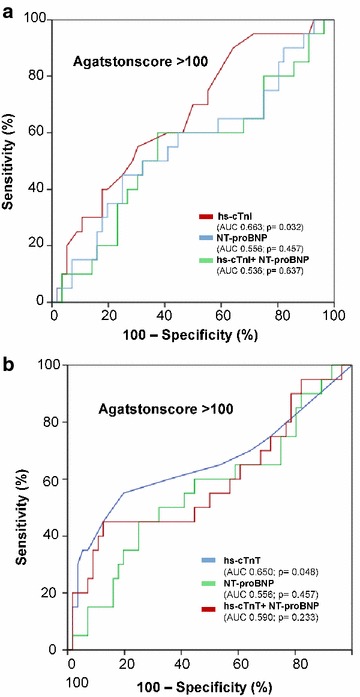
Receiver operating characteristic (ROC) curves demonstrating the capacity of hs-cTnI and hs-cTnT to discriminate significantly Agatston values greater than 100 (**a**, **b**)

As presented in Fig. 3a, b, hs-cTnI and hs-cTnT were able to discriminate Agatston score greater than 100 being assessed by CCT (hs-cTnI, AUC = 0.663; *p* = 0.032; hs-cTnT AUC = 0.650, *p* = 0.048). Combining hs-cTn plus NT-proBNP revealed no additional benefit (hs-cTnI + NT-proBNP; AUC = 0.536; *p* = 0.637; hs-cTnT + NT-proBNP, AUC = 0.590; *p* = 0.233). All other evaluated biomarkers such as NT-proBNP, uric acid, LDL, HDL, triglycerides, and creatinine did not show significant discrimination in C-statistics (data not shown).

### Odds ratio for Agatston score > 100 and Agatston score > 400

Table 4 demonstrates that patients with hs-cTnT values of ≥ 0.007 µg/l were up to five times more likely to reveal Agatston scores of greater than 100 [*n* = 11, odds ratio (OR) = 5.0; 95% CI 1.664–15.025; *p* = 0.004]. Patients with hs-cTnT values of 0.02 µg/l or greater were more than 13 times more likely to reveal Agatston scores greater than 400 (*n* = 2, OR = 13.4; 95% CI 1.545–116.233; *p* = 0.019). Noteworthily, hs-cTnI levels ≥ 5.5 ng/l were associated with the presence of Agatston scores greater than 400 (*n* = 2, OR = 8.8; 95% CI 1.183–65.475; *p* = 0.034). However, a cut-off value of 4.0 ng/l for hs-cTnI did not predict patients with Agatston score greater than 100 (*n* = 5, OR = 3.4; 95% CI 0.867–13.337; *p* = 0.079).

**Table 4:** Logistic regression analysis for evaluating the ability of hs-cTn for the detection of increased Agatston scores

Agatston score > 100	Odds ratio	95% CI	*p*	Agatston score > 400	Odds ratio	95% CI	*p*
hs-cTnT (≥ 0.007 μg/l)	5.0	(1.664–15.025)	*0.004*	hs-cTnT (≥ 0.02 μg/l)	13.4	1.545–116.233	*0.019*
hs-cTnI (≥ 4.0 ng/l)	3.4	(0.867–13.337)	0.079	hs-cTnI (≥ 5.50 ng/l)	8.8	1.183–65.475	*0.034*

Italic values indicate statistically significant *p* values (*p* < 0.05)

*CI* confidence interval, *hs-cTnT* high-sensitivity cardiac troponin T, *hs-cTNI* high-sensitivity cardiac troponin I

## Discussion

The present study evaluates the association between high-sensitivity cardiac troponins (hs-cTnI or hs-cTnT) and the Agatston score assessed by CCT in patients with a low to intermediate risk to suffer from CAD. It was demonstrated that hs-cTnI significantly and hs-cTnT numerically increased alongside increasing Agatston score (i.e., Agatston score = 0, Agatston score from 1 to 100, Agatston score from 101 to 400, and Agatston score of > 400). The Agatston score was significantly correlated with hs cardiac troponins, both in univariable and multivariable linear regression models, whereas established clinical, cardiovascular risk factors as well as known cardiovascular risk biomarkers did not reveal consistent associations with the Agatston score. Both hs-cTns were able to discriminate patients with the highest Agatston score of more than 100. Finally, increased hs cardiac troponin levels beyond a predefined cut-off value were able to predict the presence of an increased Agatston score (i.e., greater than 100 and greater than 400).

Cardiac troponins are released specifically from the myocardium due to cardiac injury during ischemic conditions. Assuming that Agatston scores are increasing with the amount of atherosclerotic burden [13–15], the increasing levels of hs-cTnI, as ascertained in the present study, might be explained due to rising probability of ischemia coming along with increasing severity of CAD. The potential ability of hs-cTnI to discriminate between the different CAC values, might allow to differentiate between patients at high risk and patients with no need for further diagnostic assessment [29]. These present findings are in accordance with a study by Olson et al. showing an association between hs-cTnI and coronary calcium score in a Danish study cohort of 1173 clinically healthy subjects [24]. It was demonstrated that hs-cTnI was able to predict Agatston values greater than 100 and showed that adding hs-cTnI to the Heart-Score led to a significant increase in C-statistics for predicting coronary artery calcification. In another large and prospective Multi-Ethnic Study of Atherosclerosis (MESA) cohort including patients free of known CAD at baseline, most coronary heart events such as myocardial infarction or death from CAD occurred in patients with an Agatston score greater than 100 [23]. Hence, the differentiation of patients with an Agatston score > 100 is important for risk stratification of coronary heart disease in asymptomatic patients. Taking the result from the C-statistics of the present study into account, hs-cTnI as well as hs-cTnT might have the potential to identify patients with Agatston score greater than 100. Whether the Agatston score might reliably detect asymptomatic patients with higher risk for adverse coronary events in the clinical setting remains unclear due to the fact that this study was performed on symptomatic patients. The lack of an additional benefit by NT-proBNP might be triggered by the study design and its smaller sample size. Former studies with a larger cohort demonstrated an association of NT-proBNP with the extent of CAC values [30, 31].

In addition to the results presented by Olson et al., the present study shows that hs-cTnI was able to discriminate Agatston values greater than 100 in C-statistics as a stand-alone biomarker. Patients with hs-cTnI values > 5.5 ng/l were up to eight times more likely to reveal an Agatston score of greater than 400. High calcium scores are being known to be associated with a high plaque burden and more advanced stages of atherosclerosis [15, 32, 33]. Therefore, it may be assumed that hs-cTnI values greater than 5.5 ng/l might reflect patients with a high plaque burden and more severe coronary artery stenoses. However, taking the data from the present study, it was not feasible to give a proper cut-off value that might predict a significant likelihood for an Agatston score greater than 100. Additionally, with only two patients having hs-cTnI values greater than 5.5 ng/l, the clinical impact and reliability of this finding need to be approved by further larger studies.

In contrast to the results presented by Korosoglu et al., showing a close correlation between hs-cTnT and the Agatston score in patients without known CAD and presenting with chest pain, the present study could not reveal a significant correlation between hs-cTnT and the Agatston score [25]. However, hs-cTnT was able to discriminate Agatston values greater than 100. Furthermore, using cut-off values for hs-cTnT of greater than 0.007 µg/l and greater than 0.02 µg/l, revealed a fivefold higher likelihood for an Agatston score greater than 100 and a 13-fold higher likelihood for an Agatston score greater than 400, respectively. With solely two patients revealing hs-cTnT values greater than 0.02 µg/l, these findings have limited impact. However, these results might indicate a proper cut-off value which should be further investigated in larger studies due to the fact that calcium scores greater than 400 are known to indicate CAD at an extensive stage and therefore imply invasive coronary angiography as the next diagnostic step [34]. However, the C-statistics regarding hs-cTnT and the discrimination of Agatston values greater than 400 in the present study did not show statistical significance, which might be the consequence of the small sample size. In a cross-sectional study including 215 Japanese men and women, Kitagawa et al. could also demonstrate that the serum concentration of hs-cTnT was associated with an increased odds ratio for an Agatston score > 100 and an Agatston score > 400 [35]. In contrast to the present study, Kitagawa et al. showed that hs-cTnT was able to discriminate Agatston values greater than 400.

## Conclusions

In conclusion, the results of the present study are in line with further studies, showing that hs-cTnI is increasing alongside with increasing Agatston score and is able to differentiate between different Agatston values and might therefore become a potentially useful biomarker in detecting patients with subclinical CAD. With the present study being performed in a symptomatic cohort, it cannot be assumed that these results can be transformed on asymptomatic patients. The data as well show that hs-cTnI might be capable of indicating the amount of calcium within coronary arteries. Regarding the likelihood of a high atherosclerotic plaque burden represented by Agatston score > 400, the present study might deliver a potential cut-off value for hs-cTnI (hs-cTnI > 5.5 ng/l). These findings should be reevaluated in larger prospective studies due to the small sample size of the current study. Furthermore, hs-cTnI as well as hs-cTnT were significantly associated with Agatston score in multivariable regression models. The C-statistics showed that hs-cTnI and hs-cTnT were both able to significantly discriminate Agatston values greater than 100. Of note, the present study could as well deliver potential cut-off values for hs-cTnT, which should be confirmed in further studies. However, hs-cTnT was not significantly differentiate between different CAC values.

## Study limitations

First, the present study is limited by its small sample size, thereby restricting propositions for the general population. To confirm the present findings, the study should be repeated prospectively including a larger sample size. Second, the study population, mostly consisting of Caucasian individuals, does not reflect a multiethnic cohort. Therefore, the results cannot be transferred to other ethnic groups or the general population. Furthermore, the present study shows lack of clinical outcome data. Taking this into account, the prognostic merit of increased cardiac troponin values within this study remains unclear. To solve this matter, longitudinal studies are needed to prove whether the selected cut-off values of hs cardiac troponin and the estimated Agatston scores might reveal an increase in risk prediction. However, regarding the study design with the inclusion of solely symptomatic patients risk prediction towards patients without any symptoms are not allowed. Furthermore, Agatston score is only validated for 120 kV. With regard to the kV range of the present study, the CAC might be less reliable.

## Abbreviations

ACS: acute coronary syndrome
AMI: acute myocardial infarction
AUC: area under the curve
CAD: coronary artery disease
CAC: coronary calcium concentration
CT: computed tomography
CCT: coronary computed tomography
CI: confidential interval
ECG: electrocardiogram
hs-cTnI: high-sensitivity coronary troponin I
hs-cTnT: high-sensitivity coronary troponin T
hs: high sensitivity
LoB: limit of blank
LoD: limit of detection
MPR: multiplanar reformat
NT-proBNP: N-terminal pro-brain natriuretic peptide
OR: odds ratio
ROC: receiver operating characteristic curve
SD: standard deviation

## References

[1] Beatty AL, Ku IA, Christenson RH, DeFilippi CR, Schiller NB, Whooley MA (2013). High-sensitivity cardiac troponin T levels and secondary events in outpatients with coronary heart disease from the Heart and Soul Study. JAMA Intern Med.

[2] Eggers KM, Lagerqvist B, Venge P, Wallentin L, Lindahl B (2007). Persistent cardiac troponin I elevation in stabilized patients after an episode of acute coronary syndrome predicts long-term mortality. Circulation.

[3] Everett BM, Brooks MM, Vlachos HE, Chaitman BR, Frye RL, Bhatt DL, Group BDS (2015). Troponin and cardiac events in stable ischemic heart disease and diabetes. N Engl J Med.

[4] Piepoli MF, Hoes AW, Agewall S, Albus C, Brotons C, Catapano AL, Cooney MT, Corra U, Cosyns B, Authors/Task Force M (2016). 2016 European guidelines on cardiovascular disease prevention in clinical practice: The Sixth Joint Task Force of the European Society of Cardiology and other societies on cardiovascular disease prevention in clinical practice (constituted by representatives of 10 societies and by invited experts): developed with the special contribution of the European Association for Cardiovascular Prevention & Rehabilitation (EACPR). Eur J Prev Cardiol.

[5] Thomas DM, Divakaran S, Villines TC, Nasir K, Shah NR, Slim AM, Blankstein R, Cheezum MK (2015). Management of coronary artery calcium and coronary CTA findings. Curr Cardiovasc Imaging Rep.

[6] Hecht HS (2015). Coronary artery calcium scanning: past, present, and future. JACC Cardiovasc Imaging.

[7] Callister TQ, Cooil B, Raya SP, Lippolis NJ, Russo DJ, Raggi P (1998). Coronary artery disease: improved reproducibility of calcium scoring with an electron-beam CT volumetric method. Radiology.

[8] Budoff MJ, Achenbach S, Blumenthal RS, Carr JJ, Goldin JG, Greenland P, Guerci AD, Lima JA, Rader DJ, Rubin GD (2006). Assessment of coronary artery disease by cardiac computed tomography: a scientific statement from the American Heart Association Committee on Cardiovascular Imaging and Intervention, Council on Cardiovascular Radiology and Intervention, and Committee on Cardiac Imaging, Council on Clinical Cardiology. Circulation.

[9] Moselewski F, O’Donnell CJ, Achenbach S, Ferencik M, Massaro J, Nguyen A, Cury RC, Abbara S, Jang IK, Brady TJ (2005). Calcium concentration of individual coronary calcified plaques as measured by multidetector row computed tomography. Circulation.

[10] Agatston AS, Janowitz WR, Hildner FJ, Zusmer NR, Viamonte M, Detrano R (1990). Quantification of coronary artery calcium using ultrafast computed tomography. J Am Coll Cardiol.

[11] Stary HC (1992). Composition and classification of human atherosclerotic lesions. Virchows Arch A Pathol Anat Histopathol.

[12] Ross R (1993). The pathogenesis of atherosclerosis: a perspective for the 1990s. Nature.

[13] Xu Y, Mintz GS, Tam A, McPherson JA, Iniguez A, Fajadet J, Fahy M, Weisz G, De Bruyne B, Serruys PW (2012). Prevalence, distribution, predictors, and outcomes of patients with calcified nodules in native coronary arteries: a 3-vessel intravascular ultrasound analysis from Providing Regional Observations to Study Predictors of Events in the Coronary Tree (PROSPECT). Circulation.

[14] Mintz GS, Pichard AD, Popma JJ, Kent KM, Satler LF, Bucher TA, Leon MB (1997). Determinants and correlates of target lesion calcium in coronary artery disease: a clinical, angiographic and intravascular ultrasound study. J Am Coll Cardiol.

[15] Greenland P, Bonow RO, Brundage BH, Budoff MJ, Eisenberg MJ, Grundy SM, Lauer MS, Post WS, Raggi P, Redberg RF (2007). ACCF/AHA 2007 clinical expert consensus document on coronary artery calcium scoring by computed tomography in global cardiovascular risk assessment and in evaluation of patients with chest pain: a report of the American College of Cardiology Foundation Clinical Expert Consensus Task Force (ACCF/AHA Writing Committee to Update the 2000 Expert Consensus Document on Electron Beam Computed Tomography) developed in collaboration with the Society of Atherosclerosis Imaging and Prevention and the Society of Cardiovascular Computed Tomography. J Am Coll Cardiol.

[16] Bonow RO (2009). Clinical practice. Should coronary calcium screening be used in cardiovascular prevention strategies?. N Engl J Med.

[17] Vliegenthart R, Morris PB (2012). Computed tomography coronary artery calcium scoring: review of evidence base and cost-effectiveness in cardiovascular risk prediction. J Thorac Imaging.

[18] Becker A, Leber A, Becker C, Knez A (2008). Predictive value of coronary calcifications for future cardiac events in asymptomatic individuals. Am Heart J.

[19] Taylor AJ, Bindeman J, Feuerstein I, Cao F, Brazaitis M, O’Malley PG (2005). Coronary calcium independently predicts incident premature coronary heart disease over measured cardiovascular risk factors: mean three-year outcomes in the prospective army coronary calcium (PACC) project. J Am Coll Cardiol.

[20] Detrano R, Guerci AD, Carr JJ, Bild DE, Burke G, Folsom AR, Liu K, Shea S, Szklo M, Bluemke DA (2008). Coronary calcium as a predictor of coronary events in four racial or ethnic groups. N Engl J Med.

[21] Haberl R, Becker A, Leber A, Knez A, Becker C, Lang C, Bruning R, Reiser M, Steinbeck G (2001). Correlation of coronary calcification and angiographically documented stenoses in patients with suspected coronary artery disease: results of 1764 patients. J Am Coll Cardiol.

[22] Knez A, Becker A, Leber A, White C, Becker CR, Reiser MF, Steinbeck G, Boekstegers P (2004). Relation of coronary calcium scores by electron beam tomography to obstructive disease in 2115 symptomatic patients. Am J Cardiol.

[23] Bittencourt MS, Blaha MJ, Blankstein R, Budoff M, Vargas JD, Blumenthal RS, Agatston AS, Nasir K (2014). Polypill therapy, subclinical atherosclerosis, and cardiovascular events-implications for the use of preventive pharmacotherapy: MESA (Multi-Ethnic Study of Atherosclerosis). J Am Coll Cardiol.

[24] Olson F, Engborg J, Gronhoj MH, Sand NP, Lambrechtsen J, Steffensen FH, Nybo M, Gerke O, Mickley H, Diederichsen AC (2016). Association between high-sensitive troponin I and coronary artery calcification in a Danish general population. Atherosclerosis.

[25] Korosoglou G, Lehrke S, Mueller D, Hosch W, Kauczor HU, Humpert PM, Giannitsis E, Katus HA (2011). Determinants of troponin release in patients with stable coronary artery disease: insights from CT angiography characteristics of atherosclerotic plaque. Heart.

[26] STAT TTh. © 2013 Roche Diagnostics. cobas^®^.

[27] STAT High Sensitive Troponin-I AS, © 2015 Abbott Laboratories.

[28] proBNP II STAT c, © 2014, Roche Diagnostics.

[29] Korley FK, George RT, Jaffe AS, Rothman RE, Sokoll LJ, Fernandez C, Falk H, Post WS, Saheed MO, Gerstenblith G (2015). Low high-sensitivity troponin I and zero coronary artery calcium score identifies coronary CT angiography candidates in whom further testing could be avoided. Acad Radiol.

[30] Kara K, Mahabadi AA, Berg MH, Lehmann N, Mohlenkamp S, Kalsch H, Bauer M, Moebus S, Dragano N, Jockel KH (2014). Predicting risk of coronary events and all-cause mortality: role of B-type natriuretic peptide above traditional risk factors and coronary artery calcium scoring in the general population: the Heinz Nixdorf Recall Study. Eur J Prev Cardiol.

[31] Abdullah SM, Khera A, Das SR, Stanek HG, Canham RM, Chung AK, Morrow DA, Drazner MH, McGuire DK, de Lemos JA (2005). Relation of coronary atherosclerosis determined by electron beam computed tomography and plasma levels of n-terminal pro-brain natriuretic peptide in a multiethnic population-based sample (the Dallas Heart Study). Am J Cardiol.

[32] Blaha MJ, Mortensen MB, Kianoush S, Tota-Maharaj R, Cainzos-Achirica M (2017). Coronary artery calcium scoring: is it time for a change in methodology?. JACC Cardiovasc Imaging.

[33] Hecht HS, Cronin P, Blaha MJ, Budoff MJ, Kazerooni EA, Narula J, Yankelevitz D, Abbara S (2017). 2016 SCCT/STR guidelines for coronary artery calcium scoring of noncontrast noncardiac chest CT scans: a report of the Society of Cardiovascular Computed Tomography and Society of Thoracic Radiology. J Thorac Imaging.

[34] Ahn SJ, Kang DK, Sun JS, Yoon MH (2013). Accuracy and predictive value of coronary computed tomography angiography for the detection of obstructive coronary heart disease in patients with an Agatston calcium score above 400. J Comput Assist Tomogr.

[35] Kitagawa N, Okada H, Tanaka M, Hashimoto Y, Kimura T, Tomiyasu K, Nakano K, Hasegawa G, Nakamura N, Fukui M (2015). High-sensitivity cardiac troponin T is associated with coronary artery calcification. J Cardiovasc Comput Tomogr.

